# Gene Co-expression Analysis Identifies Histone Deacetylase 5 and 9 Expression in Midbrain Dopamine Neurons and as Regulators of Neurite Growth via Bone Morphogenetic Protein Signaling

**DOI:** 10.3389/fcell.2019.00191

**Published:** 2019-09-13

**Authors:** Martina Mazzocchi, Sean L. Wyatt, Daniela Mercatelli, Michele Morari, Noelia Morales-Prieto, Louise M. Collins, Aideen M. Sullivan, Gerard W. O’Keeffe

**Affiliations:** ^1^Department of Anatomy & Neuroscience, University College Cork (UCC), Cork, Ireland; ^2^School of Biosciences, Cardiff University, Cardiff, United Kingdom; ^3^Department of Medical Sciences, Section of Pharmacology, University of Ferrara, National Institute of Neuroscience, Ferrara, Italy; ^4^Department of Physiology, University College Cork, Cork, Ireland; ^5^APC Microbiome Ireland, University College Cork, Cork, Ireland

**Keywords:** Parkinson’s, alpha-synuclein, degeneration, histone deacetylase, neurite growth, survival, bone morphogenetic protein, Smad

## Abstract

Parkinson’s disease is characterized by the intracellular accumulation of α-synuclein which has been linked to early dopaminergic axonal degeneration. Identifying druggable targets that can promote axonal growth in cells overexpressing α-synuclein is important in order to develop strategies for early intervention. Class-IIa histone deacetylases (HDACs) have previously emerged as druggable targets, however, it is not known which specific class-IIa HDACs should be targeted to promote neurite growth in dopaminergic neurons. To provide insight into this, we used gene co-expression analysis to identify which, if any, of the class-IIa HDACs had a positive correlation with markers of dopaminergic neurons in the human substantia nigra. This revealed that two histone deacetylases, *HDAC5* and *HDAC9*, are co-expressed with *TH, GIRK2* and *ALDH1A1* in the human SN. We further found that HDAC5 and HDAC9 are expressed in dopaminergic neurons in the adult mouse substantia nigra. We show that siRNAs targeting *HDAC5* or *HDAC9* can promote neurite growth in SH-SY5Y cells, and that their pharmacological inhibition, using the drug MC1568, promoted neurite growth in cultured rat dopaminergic neurons. Moreover, MC1568 treatment upregulated the expression of the neurotrophic factor, *BMP2*, and its downstream transcription factor, *SMAD1*. In addition, MC1568 or siRNAs targeting *HDAC5* or *HDAC9* led to an increase in Smad-dependent GFP expression in a reporter assay. Furthermore, MC1568 treatment of cultured rat dopaminergic neurons increased cellular levels of phosphorylated Smad1, which was prevented by the BMP receptor inhibitor, dorsomorphin. Dorsomorphin treatment prevented the neurite growth-promoting effects of siRNAs targeting HDAC5, as did overexpression of dominant-negative Smad4 or of the inhibitory Smad7, demonstrating a functional link to BMP signaling. Supplementation with BMP2 prevented the neurite growth-inhibitory effects of nuclear-restricted HDAC5. Finally, we report that siRNAs targeting *HDAC5* or *HDAC9* promoted neurite growth in cells overexpressing wild-type or A53T-α-synuclein and that MC1568 protected cultured rat dopaminergic neurons against the neurotoxin, MPP^+^. These findings establish HDAC5 and HDAC9 as novel regulators of BMP-Smad signaling, that additionally may be therapeutic targets worthy of further exploration in iPSC-derived human DA neurons and *in vivo* models of Parkinson’s disease.

## Introduction

Parkinson’s disease (PD) is characterized by the progressive loss of A9 midbrain dopaminergic (mDA) neurons, and the accumulation of intracellular aggregates of α-synuclein in Lewy bodies and Lewy neurites (Spillantini et al., 1997; Lees et al., 2009). α-synuclein has been linked to PD pathology by studies showing that *SNCA* mutations, including the A53T mutation, cause autosomal dominant PD (Polymeropoulos et al., 1997), and that α-synuclein is a risk factor for idiopathic PD (International Parkinson Disease Genomics Consortium, Nalls, 2011). At the cellular level, multiple studies have linked α-synuclein accumulation to axonal degeneration in PD (O’Keeffe and Sullivan, 2018). Specifically, α-synuclein overexpression in individual mDA neurons reduces neurite growth (Koch et al., 2015). There is also evidence for alterations in neurite structure (Oliveira et al., 2015), as well as decreased neurite length with increased axonal degeneration, in iPSC-derived neurons carrying an *SNCA* triplication (Lin et al., 2016) or the A53T *SNCA* mutation (Kouroupi et al., 2017). These studies implicate α-synuclein in mDA neurite degeneration and highlight the need to identify druggable targets that can prevent axonal degeneration and/or promote neurite growth in mDA neurons with an overload of α-synuclein.

Recent work has linked α-synuclein accumulation with alterations in histone acetylation, a key epigenetic mechanism that is regulated by histone deacetylases (HDACs) which are crucial for the correct expression of gene networks that regulate post-mitotic cell maintenance (Parra, 2015). Accumulation of wild-type or A53T-α-synuclein has been shown to reduce histone acetylation and to promote neurotoxicity in mDA neurons (Kontopoulos et al., 2006). Also, α-synuclein overexpression in Lund human mesencephalic (LUHMES) cells reduced histone acetylation, resulting in transcriptional deregulation and DNA damage, which could be prevented by the pan-HDAC inhibitor, sodium butyrate (Paiva et al., 2017). This suggests that HDAC inhibition may be a strategy to promote mDA neurite growth in cells carrying an α-synuclein overload.

HDACs are divided into a number of different subfamilies and those belonging to the class-IIa family (HDAC4, HDAC5, HDAC7, and HDAC9) are unique in that they can shuttle between the cytoplasm and nucleus (Harrison and Dexter, 2013; Hegarty et al., 2016b). Recent work has implicated HDAC4 as a neurotoxic mediator in *in vitro* and *in vivo* models of PD (Wu et al., 2017). Specifically, MPP^+^ treatment of cultured mDA neurons obtained from A53T α-synuclein transgenic mice led to nuclear accumulation of HDAC4, which was neurotoxic (Wu et al., 2017). In agreement with this, the class-IIa HDAC inhibitor MC1568 (Mai et al., 2005), was found to promote neurite growth in mDA neurons, and to protect against the neuritotoxic effects of MPP^+^ (Collins et al., 2015). Nuclear export of HDAC5 and HDAC9 has also been shown to be required for axonal growth in sensory neurons (Cho et al., 2013) and thalamocortical neurons (Alchini et al., 2017). However, whether class-IIa HDACs can be targeted to promote neurite growth in cells carrying α-synuclein, the mechanisms mediating these potential effects, and the relevance of this to the human midbrain, are unknown.

## Materials and Methods

### Gene Expression Analysis of the Human SN

Human SN gene expression data from healthy controls (GSE:60863) (Ramasamy et al., 2014) were analyzed using the R2: Genomics Analysis and Visualization Platform^1^. Pearson correlation analysis with a Bonferroni multiple testing correction was used to identify which HDACs were significantly co-expressed with three markers of dopaminergic neurons, *ALDH1A1*, *GIRK2*, and *TH*, and then subsequently to identify all genes that were significantly co-expressed with *HDAC5* and *HDAC9.* All gene expression data are presented as log2 expression values.

### Cell Culture

SH-SY5Y cells, a widely used cell line model of human DA neurons (Xicoy et al., 2017), were used. These cells were maintained in Dulbecco’s Modified Eagle Medium Nutrient Mixture F-12 (Sigma), supplemented with 10% fetal bovine serum (Sigma), 100 nM L-Glutamine (Sigma), 100 U/ml Penicillin (Sigma), 10 μg/ml Streptomycin (Sigma), in a humidified atmosphere containing 5% CO2 at 37°C. Primary cultures of embryonic (E) day 14 rat ventral midbrain (VM) (from the Biological Service Unit, University College Cork) were dissected as described (Hegarty et al., 2016a). All scientific procedures were performed under a license in accordance with the European Communities Council Directive (86/609/EEC) and approval by local Animal Experimentation Ethics Committee. After dissection, tissue was dissociated and neurons were plated in poly-D-lysine coated 24-well plates (Sigma) in DMEM:F12 media (Sigma) supplemented with 1% penicillin/streptomycin (Sigma), 1% L-glutamine (Sigma), 2% B27 (Invitrogen) and 1% FBS. Where indicated, cells were pre-treated for 30 min with 1 μg/ml of the BMPR1 inhibitor, dorsomorphin (Sigma). Recombinant human BMP2 was added at a final concentration of 50 ng/ml (Gibco) as in previous studies (Goulding et al., 2019). The class-IIa HDAC inhibitor MC1568 (Sigma) was added at concentrations ranging from 0.01–0.1 μM, as indicated in the figure legends.

### Plasmids and siRNA

1.5 × 10^5^ cells per well were transfected using TransIT-X2 Dynamic Delivery System (Mirus) according to the manufacturer’s instructions with various combinations of the following plasmids or siRNAs as indicated in the relevant figure legends: EGFP-alpha-synuclein-WT (Addgene plasmid # 40822^2^; RRID:Addgene_40822) and EGFP-alphasynuclein-A53T (Addgene plasmid #40823^3^; RRID:Addgene_40823) were a gift from David Rubinsztein (Furlong et al., 2000); pCMV5 DPC4 (1-514) (Smad4 dominant negative; Addgene plasmid #14040; RRID:Addgene_14040) was a gift from Joan Massague (Lagna et al., 1996). pcDNA3-Smad7 was a gift from Aristidis Moustakas (Addgene plasmid #80893^5^; RRID:Addgene_80893); GFP Cignal reporter (Qiagen CCS-017G); HDAC5 wild-type (WT) (Addgene plasmid #32213^6^; RRID:Addgene_32213) and HDAC5 S259A/S498A (Addgene plasmid #32216^7^; RRID:Addgene_32216) were gifts from Reuben Shaw (Mihaylova et al., 2011). For siRNA experiments, cells were transfected with 25 nM of commercially available *Silencer*^®^ pre-designed siRNAs targeting *HDAC4*, *HDAC5*, *HDAC7*, *HDAC9* or scrambled control siRNA (ThermoFisher).

### Quantitative Real-Time PCR (RT-qPCR)

The levels of *Hdac5* and *Hdac9* mRNAs in postnatal day (P)5 and adult (P90) VM were quantified by RT-qPCR relative to a geometric mean of mRNAs for the housekeeping enzymes glyceraldehyde phosphate dehydrogenase (*Gapdh*), succinate dehydrogenase (*Sdha*) and hypoxanthine phosphoribosyltransferase-1 (*Hprt1*) as previously described (Hegarty et al., 2017). Briefly, 5 μl of total midbrain RNA was reverse transcribed for 1 h at 45°C using the AffinityScript kit (Agilent, Berkshire, United Kingdom) in a 25 μl reaction according to the manufacturer’s instructions. 2 μl of cDNA was amplified in a 20 μl reaction volume using Brilliant III ultrafast qPCR master mix reagents (Agilent Technologies) with 150 nM of primers and 300 nM of dual-labeled (FAM/BHQ1) hybridization probes specific to each of the cDNAs (MWG/Eurofins, Ebersberg, Germany) using the Mx3000P platform (Agilent). The PCR primers were: *Hdac5* forward: 5′-TCC CTC CTA CAA ATT GCC-3′ and reverse: 5′-GGT GAT CTC AAC TGC TCT C-3′; *Hdac9* forward: 5′-CGT CTC CAT CCT ACA AGT A-3′ and reverse: 5′-CTC CTC TCT GCC ACT TTC-3′. Dual-labeled probes were: *Hdac5* 5′-FAM CCC TAT GAC AGC CGT GAT GAC T BHQ1-3′; *Hdac9* 5′-FAM TAA CCT GGA CCG CAC CTT CAA BHQ1-3′. To determine whether class-IIa HDAC inhibition regulates the expression of mRNAs encoding *BMP2* and components of the BMP signaling pathway, total RNA from SH-SY5Y cells that had been cultured for 12 h either with or without 0.1 μM MC1568 was reverse transcribed and the resulting cDNA amplified by qPCR, as described above, with primers and dual-labeled probes targeting either *BMP2*, *SMAD1*, *SMAD5, BMPR2, ACVR2A or BMPR1B.* Target mRNA levels were expressed relative to the geometric mean of the reference mRNAs, *GAPDH*, Tata binding protein (*TBP*) and β2-microglobulin (*B2M*). The primers were: *BMP2* forward: 5′-GGA GAT TCT TCT TTA ATT TAA G-3′ and reverse: 5′-ACT GCT ATT GTT TCC TAA-3′; *SMAD1* forward: 5′-CCA CTA TAA GAG AGT AGA AAG-3′ and reverse: 5′-CTG GAA AGA ATC TGG AAA-3′; *SMAD5* forward: 5′-ATG CCC AGT ATA TCC AGC-3′ and reverse: 5′-GAA GGA TCT GTG AAT CCA TC-3′; *BMPR2* forward: 5′-TGG GAA AGA AAC AAA TCT G-3′ and reverse: 5′-TGA GGA GGA AGA ATA ATC TG-3′; *ACVR2A* forward: 5′-CCA GCA TCC ATC TCT TGA-3′ and reverse: 5′-GTC GTG ATC CCA ACA TTC-3′; *BMPR1B* forward: 5′-CAC AGA AAG GAA CGA ATG-3′ and reverse: 5′-AGA GCA AAC TAC AGA CAG-3′; *GAPDH* forward: 5′-TGG TCT CCT CTG ACT TCA-3′ and reverse: 5′-GCT GTA GCC AAA TTC GTT G-3′; *TBP* forward: 5′-CTC ACA GAC TCT CAC AAC-3′ and reverse: 5′-AGG TCA AGT TTA CAA CCA A-3′; *B2M* forward: 5′-CCT GAA TTG CTA TGT GTC-3′ and reverse: 5′-CAG TGT AGT ACA AGA GAT AGA-3′. Dual labeled probes were: *BMP2* 5′-FAM-CCC ACG GAG GAG TTT ATC ACC-BHQ1-3′; *SMAD1* 5′-FAM-ACT TCC TCC TGT GCT GGT TCC-BHQ1-3′; *SMAD5* 5′-FAM-AGC CTG TTG CCT ATG AAG AGC C-BHQ1-3′; *BMPR2* 5′-FAM-TCA ATC CAA TGT CTA CTG CTA TGC-BHQ1-3′; *ACVR2A* 5′-FAM-ACA GAG CAT TGC CAT TCC AGC-BHQ1-3′; *BMPR1B* 5′-FAM-ACC TAC ACC CTA CAC TGC CTC-BHQ1-3′.

### End Point PCR

For end point PCR, two million cells per experimental condition were plated on a 6-well plate. Total RNA was isolated using the RNeasy Plus Universal Mini Kit (QIAGEN^®^) according to manufacturer’s indications. DNase I treatment was included to ensure complete removal of gDNA. RNA concentration was measured using a Thermo Scientific^TM^ NanoDrop^TM^ spectrophotometer. RNA integrity was verified by electrophoresis in a bleach agarose gel. cDNA was synthetized from 1 μg of total RNA using the LunaScript^TM^ RT SuperMix (New England Biolabs^®^ Inc.). Endpoint PCR was carried on a SureCycler 8800 Thermal Cycler (Agilent Technologies, Inc.) using OneTaq^®^ Hot Start Quick-Load^®^ (New England Biolabs^®^ Inc.) in a final volume of 20 μL. The PCR primers were: *HDAC4* forward: 5′-GAC CCA ATG CAA ACG CTG TCC GTT CC-3′ and reverse 5′-GGT GAC CGT CTC GGC TTC TTC GTT CTC-3′. *HDAC5* forward: 5′-GGC AAT GGC ACC CAG CAG GCG TTC TAC-3′ and reverse: 5′-CCA CAC CTC CTG TCC ATG CCA CGT TCA C-3′. *HDAC7* forward: 5′-CCG CTC AGC CGC CTC AAA CTG GAC AAC-3′ and *HDAC7* reverse: 5′-CCC AGC GGG CTG CAT TGG AGG AAT GA-3′. *HDAC9* forward: 5′-CGC AGT GCC ATC CCA GCT CAA TGC TTC G-3′ and reverse 5′-CCT GGT GGC TGC TGT TGG GTG GCT TTC-3′. Fifty nanograms of cDNA template were amplified for 40 cycles (95°C, 15 s + 70°C, 30 s) followed by a final extension step at 70°C for 5 min. After PCR, amplicons generated were loaded on a 1.8% agarose 1% TAE gel. Electrophoresis was run at 80 V for 45 min and bands were visualized on a UV transilluminator.

### Immunocytochemistry

Cultures were fixed for 15 min using 4% paraformaldehyde. Following 3 × 5 min washes in 10 mM PBS-T (0.02% Triton X-100 in 10 mM PBS), cultures were incubated in 5% bovine serum albumin (BSA) in 10 mM PBS-T for 1 h at room temperature. Cultures were subsequently incubated in the following primary antibodies: tyrosine hydroxylase (TH) (Millipore ab152; 1:200), α-synuclein (Millipore 36/008; 1:2000), Smad1/5/9 (Abcam ab66737 1:200) or phospho-Smad 1/5/9 (Cell Signaling 13820S 1:200), diluted in 1% BSA in 10 mM PBS at 4°C overnight. Following 3 × 5 min washes in 10 mM PBS-T, cells were incubated in 594-conjugated secondary antibodies (Invitrogen; 1:500 A11005 or A11012) in 1% BSA prior to 3 × 5 min washes and counterstained with DAPI (1:3000, Sigma). Cells were imaged using an Olympus IX71 inverted microscope. The fluorescence intensity of individual cells was measured by densitometry using Image J analysis software.

### Immunohistochemistry

Eight-week-old C57BL/6J male mice were transcardially perfused with 4% paraformaldehyde and brains were fixed overnight in 4% paraformaldehyde followed by cryoprotection in 30% sucrose. Fifty-micrometre-thick serial frozen sections were cut and processed for immunohistochemistry using primary antibodies for HDAC5 (Abcam ab55403; 1:200), HDAC9 (Abcam ab59718; 1:250) and TH (Millipore ab152; 1:1000 or Abcam ab76442; 1:1000) and DAPI (1:3000). Primary antibody binding was detected using the following secondary antibodies: AlexaFluor 594 (Life Tech A11012; 1:1000) and AlexaFluor 488 (Life Tech A11039; 1:2000). Sections were imaged using a confocal microscope.

### Neurite Growth and Statistical Analysis

Statistical analysis was performed using GraphPad Prism 6 (©2018 GraphPad Software, CA, United States). All experiments were performed in triplicate and were repeated at least three times. For analysis of neurite growth, five non-overlapping images were captured from each well in each experimental group. The measurement of neurite length was carried out using the trace function in Image J. For neurite growth measurements, ≥135 cells from at least three experiments were analyzed. All data are presented as mean ± SEM of the number of experimental replicates rather than of the number of cells. Statistical differences were analyzed using a Student’s *t*-test or one-way ANOVA as appropriate with the *post hoc* test as indicated in the figure legends.

## Results

### Co-expression Analysis of Human and Mouse SN Identifies *HDAC5* and *HDAC9* Co-expression With Multiple Markers of Midbrain Dopaminergic Neurons

**FIGURE 1:**
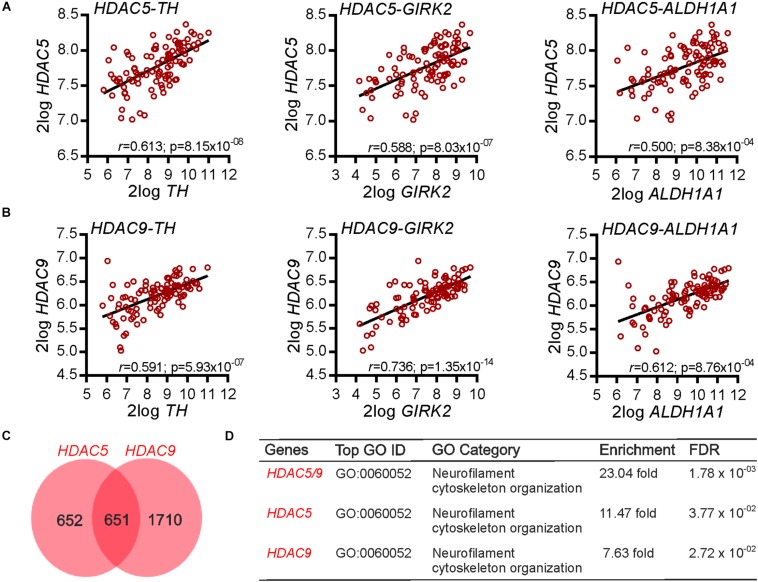
Gene co-expression analysis of *HDAC5* and *HDAC9* in the human substantia nigra. **(A,B)** Graphs showing the correlation between **(A)** *HDAC5* and **(B)** *HDAC9* and three markers of midbrain dopaminergic neurons (*TH, GIRK2/KCNJ6, ALDH1A1*) in the human substantia nigra (*n* = 101). The *r* and Bonferroni-corrected *p*-values shown on each graph. Raw data were derived from data set GSE60863 and analyzed using the R2 microarray platform. **(C)** Venn diagram showing the number of genes in the human substantia nigra displaying a multiple testing adjusted correlation of ≥0.6 with *HDAC5* (*n* = 1303) and HDAC9 (*n* = 2361), and the overlap between them (*n* = 651) in the SN. **(D)** Table showing a gene ontology (GO) enrichment analysis of these gene lists. The top category associated with each gene list is shown, along with the fold-enrichment and FDR adjusted *p* value.

We first investigated whether any class-IIa HDACs (HDAC4, 5, 7, and 9) were co-expressed with multiple markers of mDA neurons in the human SN. The rationale for this approach is that correlated patterns of gene expression can reflect a co-expression and/or a functional association (Eisen et al., 1998; Homouz and Kudlicki, 2013). To investigate this, we used available gene expression data (GSE: 60863) from the human SN (Ramasamy et al., 2014), and identified from among all genes, those class-IIa HDACs that had a significant positive correlation with the expression of three markers of mDA neurons: *TH*, *GIRK2/KCNJ6*, and *ALDH1A1.* Of the class-IIa HDACs, *HDAC5* (Figure 1A) and *HDAC9* (Figure 1B), but not *HDAC4* and *HDAC7*, were found to have a significant positive correlation with all three mDA markers. These data show that transcripts for *HDAC5* and *HDAC9* display a positive co-expression pattern with transcripts for multiple markers of mDA neurons in the human SN, suggesting that they may play a functional role in mDA neurons. To gain some insight into this possible role, we next identified all genes that had a significant positive correlation with transcripts for *HDAC5* and *HDAC9* (Figure 1C). A gene ontology (GO) analysis revealed that the GO category “*Neurofilament cytoskeletal organization*” (Figure 1D) was the top hit, suggesting that these HDACs may regulate neurite growth and or neurite maintenance.

**FIGURE 2:**
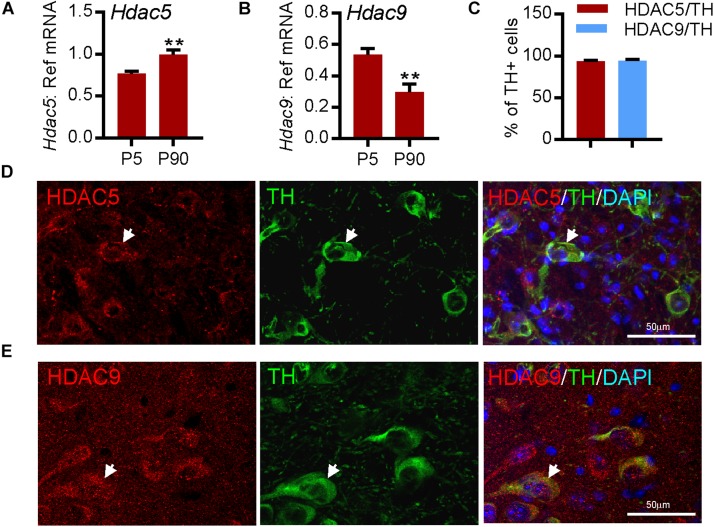
HDAC5 and HDAC9 transcripts and protein are expressed in dopaminergic neurons in mouse substantia nigra. **(A,B)** RT-qPCR showing the expression of transcripts for **(A)** *Hdac5* and **(B)** *Hdac9* in mouse midbrain at postnatal day (P)5 and P90 relative to the levels of the geometric mean of three reference mRNAs, *Gapdh*, *Sdha*, and *Hprt1*. Data are mean ± SEM from *n* = 3–6 mice at each time point. ^∗∗^*P* < 0.01, Student’s *t*-test. **(C)** Quantification of TH-immunopositive neurons in the SN that express HDAC5 or HDAC9. **(D,E)** Immunohistochemistry showing **(D)** HDAC5 (red) and **(E)** HDAC9 (red) expression in TH-positive neurons (green; arrow), colabelled with DAPI (blue), in adult mouse substantia nigra. Scale bar = 50 μm. Data are mean ± SD from *n* = 3 mice.

To confirm these findings on *HDAC5* and *HDAC9* expression in the human SN, we next used quantitative real-time PCR (RT-qPCR) to measure the levels of *Hdac5* and *Hdac9* mRNAs in mouse VM at P5 and in adulthood (P90). Both *Hdac5* (Figure 2A) and *Hdac9* (Figure 2B) were expressed at P5 and in adulthood. Immunohistochemistry confirmed the expression of HDAC5 (Figures 2C,D) and HDAC9 (Figures 2C,E), both of which were predominantly localized to the cytoplasm of almost all TH-positive mDA neurons in adult mouse SNpc (95.34 ± 1.54% for HDAC5 and 93.95 ± 2.11% for HDAC9) (Figure 2C). These data show that HDAC5 and HDAC9 are expressed by mDA neurons in the adult mouse SN.

### siRNAs Targeting *HDAC5* or *HDAC9*, but Not *HDAC4* or *HDAC7*, or Pharmacological Class-IIa Inhibition Promotes Neurite Growth in SH-SY5Y Cells and in Cultured Neurons From E14 Rat VM

**FIGURE 3:**
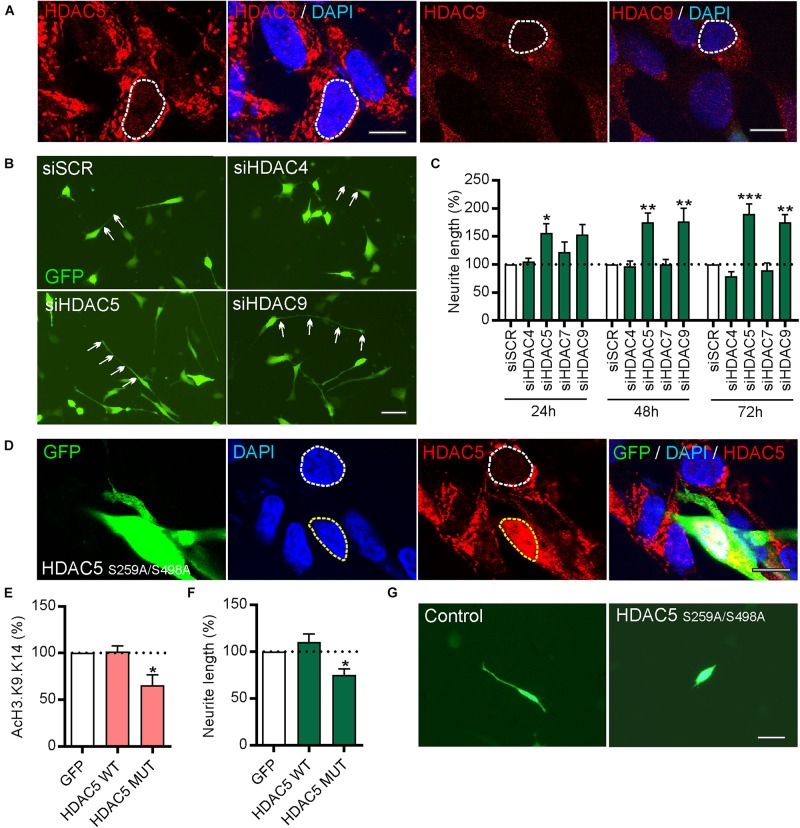
siRNAs targeting *HDAC5* or *HDAC9*, but not other class-IIa HDAC family members *HDAC4* or *HDAC7*, promote neurite growth in SH-SY5Y cells. **(A)** Representative photomicrographs of SH-SY5Y cells immunocytochemically stained for HDAC5 (red) or HDAC9 (red) with DAPI (blue). Scale bar = 10 μm. **(B)** Representative photomicrographs of SH-SY5Y cells and **(C)** graph of neurite length at 24, 48, and 72 h post-transfection with 25 nM of a scrambled siRNA (siSCR) or siRNAs against the four class-IIa HDACs (siHDAC4, siHDAC5, siHDAC7, siHDAC9). Scale bar = 50 μm. Data are mean ± SEM as percentage of the siSCR control of *n* = 3 independent experiments. ^∗^*p* < 0.05, ^∗∗^*p* < 0.01, ^∗∗∗^*p* < 0.001 vs. siSCR group; two-way ANOVA with *post hoc* Tukey’s test. **(D)** Immunocytochemistry showing nuclear localization of mutant HDAC5-S259A/S498A which is retained in the nucleus. Graphs of **(E)** AcH3-K9.K14 levels and **(F,G)** neurite length of SH-SH5Y cells at 72 h post-transfection with a control plasmid (GFP) or plasmids expressing wild-type (WT) HDAC5 or mutant HDAC5-S259A/S498A. Scale bar = 100 μm. Data are presented as the mean ± SEM as a percentage of the GFP control of *n* = 3 independent experiments. ^∗^*p* < 0.05 vs. Control; one-way ANOVA with *post hoc* Fishers LSD test.

To study the function of these HDACs, human SH-SY5Y cells were first used as these express all four class-IIa HDACs (Figure 3A and Supplementary Figures 1A,B) and as they are often used as a cell line model of human DA neurons for initial functional characterization of new molecular targets albeit with some limitations (Xicoy et al., 2017). Given the gene co-expression analysis results (Figures 1C,D), neurite growth was used as a functional readout which allowed the effects of HDAC knockdown in individual cells to be examined. To do this, SH-SY5Y cells were transfected with a plasmid expressing GFP to identify transfected cells, together with 25 nM of either a scrambled siRNA, or siRNAs against *HDAC5, HDAC9*, *HDAC4*, or *HDAC7*. Quantification of neurite growth in GFP-expressing cells revealed that only siRNAs against *HDAC5* or *HDAC9* led to significant increases in neurite growth which were progressively greater over time at 24, 48 and 72 h (Figures 3B,C). Densitometry confirmed that these siRNAs reduced HDAC protein levels (Supplementary Figure 2). In support of these findings, we also transfected SH-SY5Y cells with either a control plasmid, or a plasmid expressing either wild-type HDAC5 or a HDAC5 mutant that is retained in the nucleus (HDAC5-S259A/S498A), where it maintains deacetylase activity (Cho et al., 2013; Figure 3D). Cells expressing nuclear-restricted HDAC5 had significantly lower levels of acetylated histone 3 (AcH3)-K9.K14 (Figure 3E) and of neurite growth (Figures 3F,G) compared to controls. These data indicate that neurite growth in SH-SY5Y cells is negatively regulated by class-IIa *HDAC5* or *HDAC9*, and that cytosolic export of HDAC5 is required to prevent this process.

**FIGURE 4:**
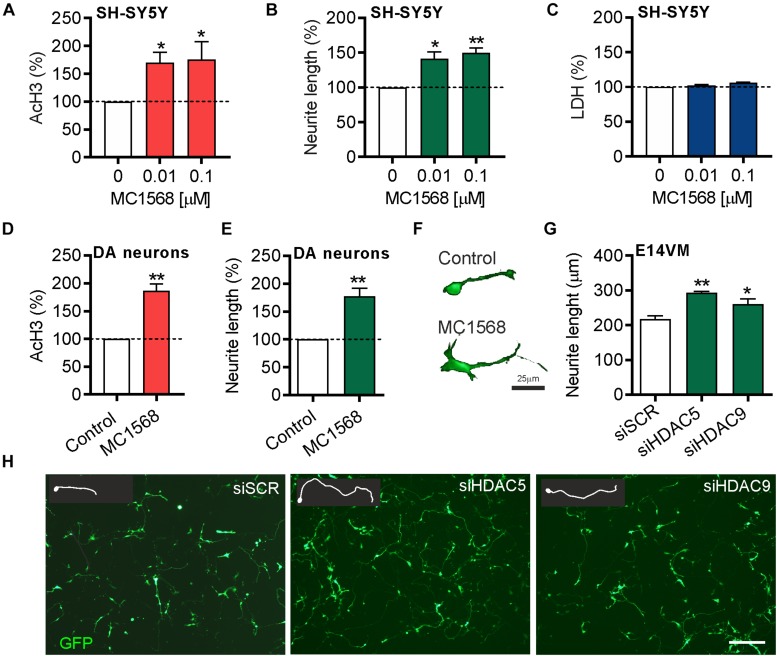
Beneficial effects of pharmacological or siRNA-mediated inhibition of *HDAC5* or *HDAC9* in SH-SY5Y cells and dopamine neurons in E14 rat VM primary cultures. Graphs showing **(A)** the relative levels of acetylated histone 3 (Ach3), **(B)** neurite length and **(C)** lactate dehydrogenase (LDH) levels as a measure of cell viability in SH-SY5Y cells treated with 0.01 or 0.1 μM MC1568 for 72 h. **(D,E)** Graphs showing **(D)** the relative levels of acetylated histone 3 (Ach3) and **(E,F)** neurite length in TH-positive neurons in primary cultures of E14 rat VM treated with 0.01 μM MC1568 for 24 h. **(G)** Graph showing neurite length and **(H)** representative photomicrographs of primary cultures of E14 rat VM at 24 h post-transfection with 25 nM of a scrambled siRNA (siSCR) or siRNAs against HDAC5 (siHDAC5) or HDAC9 (siHDAC9). Scale bar = 50 μm. Insert are tracing of individual neurons in the image. Data are mean ± SEM of *n* = 3 independent experiments. ^∗^*p* < 0.05, ^∗∗^*p* < 0.01 vs. siSCR group; Student’s *t*-test or one-way ANOVA with *post hoc* Fishers LSD test.

To provide support for these findings, we also used the class-IIa pharmacological inhibitor MC1568, which inhibits both HDAC5 and HDAC9 (Mai et al., 2005). SH-SY5Y cells were treated for 72 h with 0.01 or 0.1 μM of MC1568. Quantification of the levels of AcH3-K9.K14 (as a readout of HDAC inhibition) showed a significant increase in histone acetylation after MC1568 treatment (Figure 4A). Similar to the effects of HDAC5 and HDAC9 inhibition, treatment with MC1568 also led to an increase in neurite growth (Figure 4B), without any adverse effects on cell survival, as measured using an LDH assay (Figure 4C). To confirm the relevance of these findings in cultured DA neurons, we treated primary cultures of E14 rat VM with 0.01 μM of MC1568 for 24 h. Again we found an increase in AcH3 (Figure 4D), and also an increase in neurite length in DA neurons in these cultures (Figures 4E,F). To confirm that a similar increase was also seen with the siRNA, we transfected primary cultures of E14 rat VM with siRNAs against *Hdac5* or *Hdac9* or a scrambled control. In agreement with the SH-SY5Y data, siRNAs targeting *Hdac5 or Hdac9* increased neurite growth in cultured neurons (siSCR = 217.3 ± 9.8 μm vs. siHDAC9 = 293.1 ± 3.7 μm and siHDAC9 = 260 ± 16.4 μm) (Figures 4G,H).

### Bioinformatics Analysis Links HDAC5/9 to the Bone Morphogenetic Protein Pathway

To gain insights into the molecular mechanisms that underlie the neurite growth-promoting effects of *HDAC5* and *HDAC9* knockdown, we first analyzed gene expression data from a previously published microarray study that profiled gene expression in primary cultures of E14 mouse VM after treatment with the pan-HDIs trichostatin A, valproic acid or sodium butyrate for 48 h (Forgione and Tropepe, 2012). This analysis identified 127 genes that were significantly upregulated by all three HDIs (Forgione and Tropepe, 2012). We performed a gene ontology (GO) enrichment analysis^8^ which revealed a statistically significant overrepresentation of genes associated with GO categories related to multiple aspects of axon development (data not shown), and to the bone morphogenetic protein (BMP) signaling pathway (12.44-fold enrichment; adj. *p* = 0.0127).

We next examined data from a previous report that performed an unbiased analysis of HDAC5 genomic binding sites using ChIP-seq in mouse striatal neurons (Taniguchi et al., 2017). We performed a KEGG pathway analysis of HDAC5-associated target genes which revealed a significant enrichment of genes associated with the ‘*TGF-beta signaling pathway*’ (KEGG ID: 04350; adj. *p* = 6.67 × 10^–05^). Among the list of genes associated with this pathway was *BMP2*, which is a neurotrophic factor for mDA neurons (Goulding et al., 2019). Collectively, these data suggest that *HDAC5* and *HDAC9* inhibition may regulate *BMP2* expression and/or BMP signaling.

### Selective Class-IIa Inhibition Increases BMP Signaling in SH-SY5Y Cells and in Cultured DA Neurons From E14 Rat VM

**FIGURE 5:**
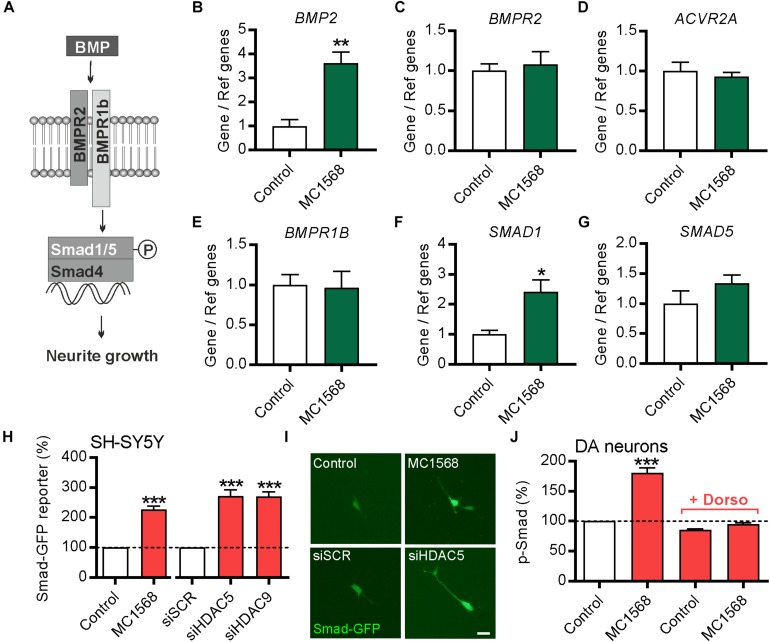
Class-IIa HDAC inhibition increases *BMP2* and *SMAD1* expression in SH-SY5Y cells. **(A)** Schema of the BMP pathway. **(B–G)** RT-qPCR data showing **(B)** *BMP2*, **(C)** *BMPR2*, **(D)** *ACVR2A*, **(E)** *BMPR1B*, **(F)** *SMAD1* and **(G)** *SMAD5* mRNA levels relative to the levels of the geometric mean of three reference mRNAs, *GAPDH*, *TBP* and *B2M*, in SH-SY5Y cells treated for 12 h with 0.1 μM of the class-IIa HDAC inhibitor MC1568. Data are mean ± SEM from *n* = 4 independent experiments expressed as fold change relative to the control. **(H)** Graph and **(I)** representative photomicrographs showing the expression of GFP (as a readout of Smad-dependent transcription) in SH-SY5Y cells transfected with a Smad-GFP reporter construct and co-transfected with either a scrambled siRNA, or siRNAs against *HDAC5* or *HDAC9* or treated with MC1568 for 72 h. **(J)** Graph showing the relative levels of pSmad1/5/8 in TH-positive neurons in primary cultures of E14 rat VM at 24 h post-treatment with 0.01 μM MC1568. Scale bar = 50 μm. Data are mean ± SEM from *n* = 3 independent experiments expressed as fold change relative to the control. ^∗^*p* < 0.05, ^∗∗^*p* < 0.01, ^∗∗∗^*p* < 0.001 vs. Control; Student’s *t*-test or one-way ANOVA with *post hoc* Tukey’s test as appropriate).

To test this, SH-SY5Y cells were treated for 12 h with 0.1 μM of the class-IIa pharmacological inhibitor MC1568 (Mai et al., 2005). The expression of *BMP2* and multiple components of the BMP pathway, including the BMP receptors (*BMPR2, ACVR2A* and *BMPR1B*) and of the R-Smads transcription factors (*SMAD1* and *SMAD5*) was examined by RT-qPCR (Figure 5A). A pharmacological approach was used rather than siRNA due to the low transfection efficiency of SH-SY5Y cells (∼25%). MC1568 treatment led to a significant increase in the expression of transcripts for *BMP2* and its transcription factor *SMAD1*, without affecting the expression of other components of the BMP-Smad pathway (Figures 5B–G). To provide further evidence that MC1568 treatment modulates BMP signaling, we next analyzed BMP-dependent transcription. To do this, SH-SY5Y cells were transfected with a Smad-GFP reporter construct in which GFP expression is under the control of a Smad-responsive minimal promoter, and were co-transfected with either a scrambled siRNA, or siRNAs against *HDAC5* or *HDAC9*, or were treated with MC1568. The level of GFP fluorescence was quantified at 72 h, revealing that siRNAs against *HDAC5* or *HDAC9*, or treatment with MC1568, led to significant increases in GFP expression, indicating a Smad-dependent transcriptional response (Figures 5H,I).

To confirm the relevance of this for cultured DA neurons, we treated cultures of E14 rat VM with MC1568 with or without dorsomorphin, a small molecule that blocks BMP signaling (Yu et al., 2008), and quantified the levels of pSmad1/5/8 in TH-positive neurons in these cultures. This analysis revealed that MC1568 treatment lead to a significant increase in the levels of pSmad1/5/8 in DA neurons, which was blocked by dorsomorphin (Figure 5J). Collectively, these data suggest that the BMP-Smad signaling pathway may mediate the neurite growth-promoting effects of *HDAC5* and *HDAC9* inhibition.

### *HDAC5* siRNA or MC1568 Promotes Neurite Growth Through the BMP-Smad Signaling Pathway

**FIGURE 6:**
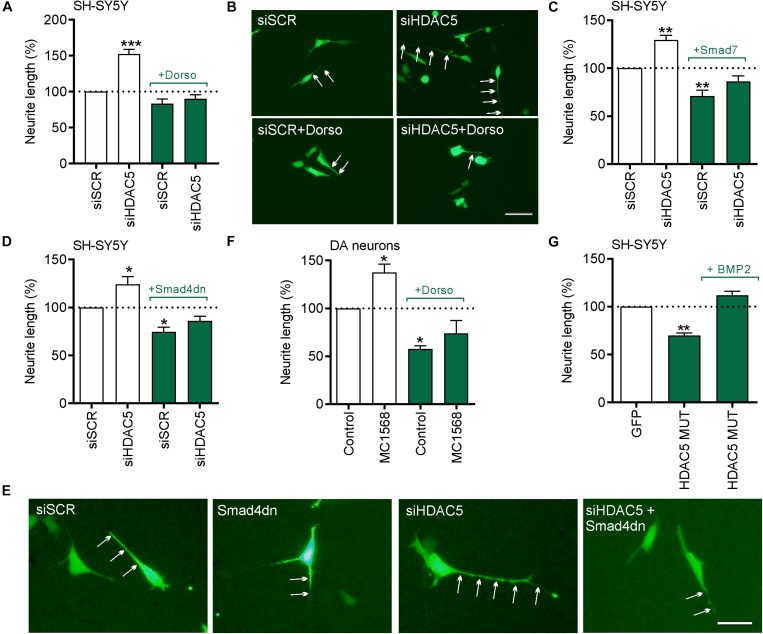
The neurite growth-promoting effects of HDAC5 inhibition require BMP-Smad signaling. **(A–E)** In all experiments cells were transfected with 25 nM of a scrambled siRNA (siSCR) or siRNAs against HDAC5 (siHDAC5) and neurite length was analyzed at 24 h. **(A)** Quantification of neurite length per cell **(B)** representative photomicrographs when co-treated with 1 μg/ml of the BMPR1 inhibitor, dorsomorphin. Neurites are indicated by white arrows. Scale bar = 50 μm. **(C,D)** Quantification of neurite length per cell and **(E)** representative photomicrographs of cells co-transfected with plasmids overexpressing **(C)** the inhibitory I-Smad, Smad7 or **(D,E)** Smad4 dominant negative (Smad4dn). **(F)** Graph showing the neurite length of TH-positive neurons in primary cultures of E14 rat VM at 24 h post-treatment with 0.01 μM MC1568. **(G)** A rescue experiment in which the cells were transfected with the nuclear-restricted HDAC5 mutant and cultured with or without 50 ng/ml BMP2. Data mean ± SEM as a percentage of the control of *n* = 3–4 independent experiments. ^∗^*p* < 0.05, ^∗∗^*p* < 0.01, ^∗∗∗^*p* < 0.01 vs. siSCR group alone; one-way ANOVA with *post hoc* Tukey’s test.

We next tested the hypothesis that the BMP-Smad signaling pathway may mediate the neurite growth-promoting effects of *HDAC5* inhibition. To do this, SH-SY5Y cells were transfected with a GFP expression plasmid together with 25 nM of either a control siRNA or siRNAs against *HDAC5*, and cultured for 24 h with or without dorsomorphin. Transfection with *HDAC5* siRNA led to a significant increase in neurite growth compared to the scrambled control (siSCR), which was not seen when siHDAC5 cells were also treated with dorsomorphin (siSCR = 49.74 ± 1.37 μm vs. siHDAC5 = 76.40 ± 1.82 μm vs, siHDAC5 + dorsomorphin = 44.05 ± 1.39 μm) (Figures 6A,B).

To further confirm the potential involvement of the BMP-Smad pathway, we transfected SH-SY5Y cells with 25 nM of either a control siRNA or *HDAC5* siRNA, and co-transfected them with either a control plasmid or a plasmid expressing the inhibitory SMAD7 (Figure 6C) or dominant-negative SMAD4 (SMAD4dn) (Figures 6D,E). Transfection with *HDAC5* siRNA led to a significant increase in neurite growth compared to the scrambled control, which was not seen when co-transfected with expression plasmids for SMAD7 or SMAD4dn (Figures 6C–E). To confirm the relevance of this for cultured DA neurons, we treated primary cultures of E14 rat VM with MC1568 with or without dorsomorphin for 24 h. We found that treatment with MC1568 led to a significant increase in neurite growth of DA neurons, that was prevented by co-treatment with dorsomorphin (Figure 6F).

We next performed a rescue experiment to determine if supplementation with BMP2 could rescue the reduction in neurite growth seen in cells expressing nuclear-restricted HDAC5. To do this, SH-SY5Y cells were transfected with the plasmid expressing HDAC5-S259A/S498A and cultured with or without 50 ng/ml BMP2 for 24 h. Quantification of neurite growth revealed a significant decrease in HDAC5-S259A/S498A-expressing cells compared to controls (100% vs. 69.75 ± 2.62%; *p* < 0.01) (Figure 6G). In contrast, we found that BMP2 treatment of HDAC5-S259A/S498A-expressing cells normalized neurite growth to control levels (100% vs. 111.70 ± 4.35%; not significant) (Figure 6G). Collectively, these data show that the BMP-Smad signaling pathway mediates the growth-promoting effects of *HDAC5* inhibition.

### *HDAC5* and *HDAC9* siRNA Promotes Neurite Growth in Cells Overexpressing Wild-Type or A53T α-Synuclein and in Cultured DA Neurons Treated With MPP^+^

**FIGURE 7:**
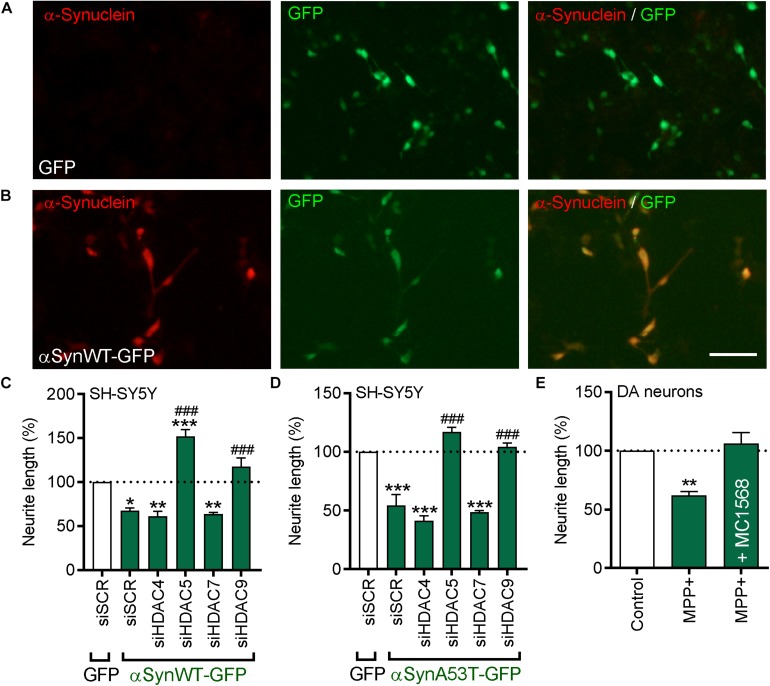
Beneficial effects of pharmacological or siRNA-mediated inhibition of *HDAC5* or *HDAC9* in cells overexpressing wild-type or A53T α-synuclein, or treated with MPP^+^. **(A,B)** Representative photomicrographs of SH-SY5Y cells transfected with **(A)** GFP or **(B)** an expression plasmid expressing GFP-tagged wild-type α-synuclein (αSynWT-GFP) and immunocytochemically stained for α-synuclein (red). **(C,D)** Graphs of neurite length of SH-SY5Y cells at 72 h post-transfection with 25 nM of a scrambled siRNA (siSCR) or siRNAs against the four class-IIa HDACs (siHDAC4, siHDAC5, siHDAC7, siHDAC9) and co-transfected with a GFP control plasmid or a plasmid expressing **(C)** αSynWT-GFP or **(D)** αSynA53T-GFP. Data are presented as the mean ± SEM as a percentage of the GFP control of *n* = 3 independent experiments. ^∗^*p* < 0.05, ^∗∗^*p* < 0.01, ^∗∗∗^*p* < 0.001 vs. Control; ###*p* < 0.001 vs. siSCR plus WT or A53T α-synuclein: One-way ANOVA with Tukey’s *post hoc* test. **(E)** Graphs of neurite length of TH-positive neurons in primary cultures of E14 rat VM at 24 h post-treatment with 1 mM MPP+ with or without 0.01 μM of MC1568. Data are presented as mean ± SEM as a percentage of the control of *n* = 3 independent experiments. ^∗∗^*p* < 0.01, vs. Control; One-way ANOVA with Fishers LSD *post hoc* test.

At the cellular level, a pathological hallmark of PD is the accumulation of intracellular α-synuclein. Therefore, we next examined whether inhibition of class-IIa HDACs could promote neurite growth in cells overexpressing wild-type (WT) or A53T-α-synuclein. SH-SY5Y cells were transfected with either a control-GFP plasmid, or a plasmid expressing either GFP-tagged WT (αSynWT-GFP) or A53T α-synuclein (αSynA53T-GFP) (Figures 7A,B). Cultures were also co-transfected with siRNA against each of the four class-IIa HDACs and then cultured for 72 h. Quantification of the neurite lengths of transfected cells revealed that overexpression of WT-α-synuclein resulted in a significant decrease in neurite length when compared to controls (23.76 ± 0.92 μm vs. 35.58 ± 0.92 μm; *p* < 0.001) (Figure 7C). Co-transfection of WT-α-synuclein-overexpressing cells with siRNAs against *HDAC5* or *HDAC9*, led to significant increases in neurite growth compared to the groups with the scrambled siRNA and the control plasmid, and also to the groups with the scrambled siRNA plus WT-α-synuclein (siSCR + WT-α-synuclein = 23.76 ± 0.92 μm vs. siHDAC5 + WT-α-synuclein = 53.73 ± 1.35 μm or siHDAC9 + WT-α-synuclein = 42.05 ± 1.17 μm; *p* < 0.001) (Figure 7C). Transfection with siRNAs against *HDAC4* and *HDAC7* did not affect neurite growth in cells expressing WT-α-synuclein (Figure 7C).

Similarly, overexpression of A53T-α-synuclein also resulted in a significant decrease in neurite length when compared to controls (17.46 ± 0.89 μm vs. 31.40 ± 0.09 μm; *p* < 0.001) (Figure 7D). siRNAs against *HDAC5* and *HDAC9*, but not *HDAC4* and *HDAC7*, also led to significant increases in neurite growth compared to the group transfected with the scrambled siRNA or the control plasmid, and compared to the group transfected with the scrambled siRNA plus WT-α-synuclein (siSCR + A53T-α-synuclein = 17.46 ± 0.89 μm vs. siHDAC5 + A53T-α-synuclein = 39.54 ± 1.37 μm or siHDAC9 + WT-α-synuclein = 35.72 ± 1.49 μm; *p* < 0.001) (Figure 7D). Collectively, these data show that siRNAs targeting *HDAC5* or *HDAC9* can promote neurite growth in cells overexpressing WT- or A53T-α-synuclein.

We next sought to confirm the relevance of this for cultured DA neurons. Given the difficulties in transfecting sufficient amounts of DA neurons, we treated primary cultures of E14 rat VM with the neurotoxin MPP^+^ and cultured them for 24 h with or without 0.01 μM MC1568. Treatment with MPP^+^ lead to a significant decrease in the neurite length of TH-positive neurons that was not seen in cultures co-treated with MC1568 (Figure 7E). Collectively, these data suggest that class-IIa HDAC inhibition, likely through inhibition of HDAC5 and HDAC9, can protect cells against α-synuclein- and MPP^+^-induced degeneration.

## Discussion

Gene co-expression analysis is a recently developed approach for the analysis of cellular functional organization by identifying coordinated patterns of co-expressed genes (van Dam et al., 2018). In the present study we have used gene-co-expression analysis of the human SN to show a significant correlation between *HDAC5* and *HDAC9* and multiple markers of human dopaminergic neurons. We confirmed the gene co-expression pattern by showing that HDAC5 and HDAC9 were expressed in dopaminergic neurons in the adult mouse SN.

We next transfected SH-SY5Y cells with siRNAs targeting each member of the class-IIa HDAC family. We found that siRNAs targeting HDAC5 and HDAC9, but not HDAC4 and HDAC7, led to a significant increase in neurite growth. This is consistent with a previous report showing that the class-IIa HDAC inhibitor MC1568 promotes dopaminergic and sympathetic axon growth (Collins et al., 2015). Our current findings show that the positive effects of class-IIa HDAC inhibition on axon growth are mediated through the inhibition of HDAC5 and HDAC9, rather than of HDAC4 and HDAC7. We further found that overexpression of nuclear-restricted HDAC5, but not of wild-type HDAC5, led to a significant decrease in neurite growth. These findings are fully consistent with previous reports showing that axon injury in peripheral sensory neurons resulted in nuclear export of HDAC5, which promoted axon regeneration both *in vitro* and *in vivo* (Cho et al., 2013). Moreover, previous studies have implicated HDAC9 as a negative regulator of reinnervation of mouse skeletal muscle (Macpherson et al., 2015), and shown that nucleo-cytoplasmic translocation of HDAC9 plays a critical role in activity-dependent thalamocortical axon branching (Alchini et al., 2017), showing that a cytoplasmic localization of HDAC5 promotes neurite growth. Immunohistochemical staining of the adult mouse SN confirmed a predominantly cytoplasmic localization for HDAC5 and HDAC9, suggesting that this may create a permissive environment for dopaminergic axon maintenance in adulthood. However, this will require further investigation.

In this study, we also examined potential mechanisms by which inhibition of HDAC5 and HDAC9 may promote neurite growth. A previous study using unbiased ChIP-seq analysis of HDAC5 genomic binding sites revealed an association between HDAC5 and the BMP2 promoter (Taniguchi et al., 2017). BMP2 has been proposed as a neuroprotective factor for the treatment of PD (O’Keeffe et al., 2017; Goulding et al., 2019). The association reported in the Taniguchi study suggests that HDAC5 may regulate BMP2 expression. In agreement with this, we found that pharmacological inhibition of class-IIa HDACs, using the selective classIIa HDAC inhibitor MC1568, led to a significant increase in *BMP2* and *SMAD1* expression in SH-SY5Y cells. More recently, BMP2 and BMPRs have been found to be co-expressed with multiple markers of dopaminergic neurons in the adult human SN (Goulding et al., 2019), suggesting that BMPs may contribute to the maintenance of these neurons in adulthood. Supporting evidence for this comes from a study of BMPR2 dominant negative (BMPR2DN) mice (Chou et al., 2008). Adult BMPR2DN mice displayed a 20% loss in the number of mDA neuron neurons, and a ∼90% loss of striatal innervation (Chou et al., 2008). The expression of HDAC5 and HDAC9 in adult dopaminergic neurons suggests that HDAC5 and HDAC9 may have potential as new therapeutic targets for stimulating BMP2 and SMAD1 expression *in vivo* in the adult brain.

Since HDAC inhibition resulted in increased BMP2 expression in SH-SY5Y cells, and activated Smad dependent gene transcription, we hypothesized that the canonical BMP-Smad pathway may underlie the neurite growth-promoting effects of HDAC5 and HDAC9 inhibition. In support of this, BMP2 has previously been shown to promote neurite growth in both SH-SY5Y cells (Hegarty et al., 2013) and cultured rat dopaminergic neurons (Hegarty et al., 2014) through the canonical BMPR-Smad pathway. BMPs have also been shown to be important in the regulation of dopaminergic development *in vivo* (Jovanovic et al., 2018), and knockout of a negative regulator of the BMP-Smad pathway leads to striatal hyperinnervation during development (Hegarty et al., 2017). In support of a role for regulation of BMP-Smad pathway by HDAC5 and HDAC9, we found that inhibition of the BMP-Smad pathway using either of three complementary strategies - treatment with the BMPR1 inhibitor dorsomorphin, overexpression of Smad4dn or overexpression of the inhibitory Smad7 - prevented the neurite growth-promoting effects of HDAC siRNAs. In support of a link between BMP and HDACs, previous work has also shown that HDACs act to suppress BMP-promoted astrogliogenesis in the embryonic forebrain (Scholl et al., 2012) and that HDAC-dependent transcriptional repression of Bmp-7 potentiates TGF-β mediated renal fibrosis (Manson et al., 2014). Collectively, these data suggest that HDAC5 and HDAC9 may act as negative regulators of the BMP-Smad pathway and that they act to limit the extent of neurite growth.

Finally, we found that HDAC5 and HDAC9 siRNA could promote neurite growth in SH-SY5Y cells overexpressing either wild-type or A53T-α-synuclein. α-synuclein has previously been shown to bind directly to histones and to reduce the level of acetylated histone 3 in cultured cells (Kontopoulos et al., 2006; Paiva et al., 2017). Moreover a recent transcriptome analysis of Lund Human Mesencephalic (LUHMES) cells showed that treatment with the pan-HDAC inhibitor sodium butyrate rescued α-synuclein-induced transcriptional changes (Paiva et al., 2017). The authors proposed that future studies will be crucial to define novel possible targets for intervention in PD. Here we suggest that HDAC5 and HDAC9 may be such targets. Furthermore, since inhibition of HDAC5 and HDAC9 upregulated BMP2 expression in this study, it is worth noting that BMP2 can promote neurite growth in both SH-SY5Y cells and in cultured rat midbrain neurons overexpressing α-synuclein (Goulding et al., 2019).

In agreement with these findings, we also report that class-IIa HDI can promote neurite growth of cultured DA neurons and protect them against MPP^+^-induced degeneration. Pan-HDAC inhibitors such as sodium butyrate, valproic acid and SAHA have been shown to protect cells from apoptosis and mitochondrial fragmentation induced by MPP^+^ treatment (Kidd and Schneider, 2010). Given the lack of specificity of these HDIs, our data suggest that HDAC5 and HDAC9 inhibition may, at least in part, underlie the beneficial effects of HDAC inhibition in DA neurons. These findings suggest that HDAC5 and HDAC9 inhibition may have therapeutic potential to protect against α-synuclein-induced degeneration. In agreement with this proposal of targeting individual HDACs, one study has shown that pharmacological inhibitors of, or siRNA against, the Class-III HDAC SIRT2 rescued α-synuclein-induced toxicity and modified inclusion morphology in a cellular model of PD (Outeiro et al., 2007). In contrast however, a recent study has shown that the Class III HDI nicotinamide exacerbated neurodegeneration in the lactacystin rat model of PD (Harrison et al., 2019). This highlights the importance of targeting specific HDACs in any therapeutic application.

In summary, we show that inhibition of HDAC5 and HDAC9 promotes neurite growth through regulation of the BMP-Smad signaling pathway. These findings suggest that HDAC5 and HDAC9 may be novel therapeutic targets worthy of further exploration in strategies aimed at axonal protection against α-synuclein-induced damage in iPSC-derived human DA neurons and *in vivo* models of PD.

## Data Availability

The raw data supporting the conclusions of this manuscript will be made available by the authors, without undue reservation, to any qualified researcher.

## Ethics Statement

The animal study was reviewed and approved by the Animal Ethics and Experimentation Committee (AEEC), University College Cork, Cork, Ireland.

## Author Contributions

MMa carried out the cell culture experiments. MMa and NM-P carried out the PCR. MMa, DM, and MMo carried out the *in vivo* immunohistochemistry. SW carried out the RT-qPCR analysis. MMa, NM-P, SW, LC, AS, and GO’K analyzed the data, prepared the figures, and wrote the manuscript. All authors edited the final manuscript. SW, MMo, LC, AS, and GO’K designed the study and supervised the work.

## Conflict of Interest Statement

The authors declare that the research was conducted in the absence of any commercial or financial relationships that could be construed as a potential conflict of interest.

## Footnotes

1 https://hgserver1.amc.nl/cgi-bin/r2/main.cgi

2 http://n2t.net/addgene:40822

3 http://n2t.net/addgene:40823

5 http://n2t.net/addgene:80893

6 http://n2t.net/addgene:32213

7 http://n2t.net/addgene:32216

8 http://geneontology.org/
