# Supplementary material for: Gene Co-expression Analysis Identifies Histone Deacetylase 5 and 9 Expression in Midbrain Dopamine Neurons and as Regulators of Neurite Growth via Bone Morphogenetic Protein Signaling
Source: Front Cell Dev Biol. 2019 Sep 13;7:191. doi: 10.3389/fcell.2019.00191 (PMC6753186; doi:10.3389/fcell.2019.00191)
Supplement: Supplementary file 1 [file Table_1.DOCX]

Supplementary Material


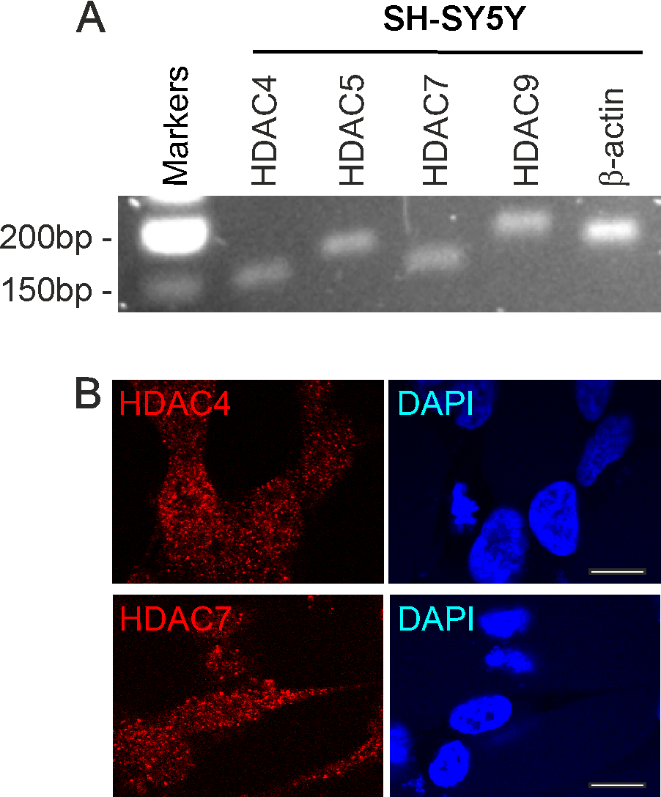


**Supplementary Figure 1: Class-IIa HDAC expression in SH-SY5Y cells.**

(A) RT-PCR showing the expression of HDAC4, 5, 7 and HDAC9 in SH-SY5Y cells along with b-actin as a control. (B) Representative photomicrographs of SH-SY5Y cells immunocytochemically stained for the HDAC4 (red) or HDAC7 (red) with DAPI (blue). Scale bar = 10 μm.


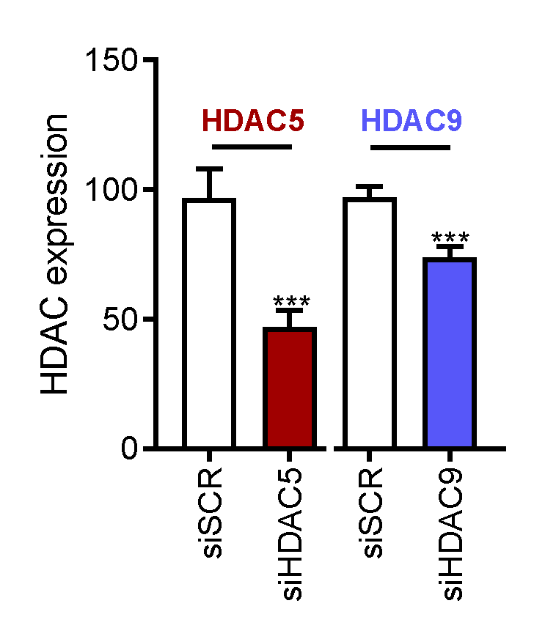


**Supplementary Figure 2: Reduced HDAC expression by HDAC siRNA in SH-SY5Y cells.**

Graph showing the quantification of HDAC5 and HDAC9 expression in SH-SY5Y cells, 24h post-transfection with 25nM of siRNAs against HDAC5 (siHDAC5) or HDAC9 (siHDAC9) compared to cells transfected with a scrambled control siRNA (siSCR). Data are presented as the mean ± SEM as a percentage of the GFP control of *n* = 3 independent experiments. **p* < 0.05, ***p* < 0.01, ****p* < 0.001 vs Control; ###*p* < 0.001 vs siSCR plus WT or A53T α-synuclein: One-way ANOVA with Tukey’s *post hoc* test.
